# Lipidomic Analysis Reveals the Anti-Obesity and Hepatoprotective Effects of Flavonoid Mimetic Components in Adzuki Beans on High-Fat Diet-Induced Obese Mice

**DOI:** 10.3390/foods14183191

**Published:** 2025-09-13

**Authors:** Jiayu Zhang, Xiujie Jiang, Qingpeng Xu, Weidong Li, Dongjie Zhang

**Affiliations:** 1 College of Food Science and Engineering, Heilongjiang Bayi Agricultural University, Daqing 163319, China; zjy_1997@outlook.com (J.Z.);; 2 Supervision, Inspection and Testing Center for Agricultural Products and Processed Products, Ministry of Agriculture and Rural Affairs, Daqing 163319, China; 3 National Coarse Cereals Engineering Research Center, Heilongjiang Bayi Agricultural University, Daqing 163319, China

**Keywords:** red adzuki bean (*Vigna angularis*), flavonoids, anti-obesity, high-fat diet, lipid metabolomics

## Abstract

Obesity and overweight have increasingly posed a serious challenge to public health security. This study systematically evaluated the reversal and regulatory effects of a composite flavonoid component mimicking the composition of adzuki bean flavonoids on high-fat diet (HFD)-induced obesity, related lipid metabolism disorders, and impaired liver function, based on lipid metabolomics and an HFD-induced obese mouse model. The results demonstrated that sustained HFD intake led to significant weight gain, increased adiposity index, dyslipidemia, and altered brown adipose tissue (BAT) cell status in mice, while also exerting adverse effects on hepatic lipid deposition and the lipid metabolic profile associated with liver fibrosis. Intervention with an adzuki bean flavonoid mimic (ABFM) effectively prevented further weight gain and ameliorated abnormal expression of serum lipid and liver function-related indicators. Furthermore, we found that ABFM alleviated HFD-induced liver damage and mitigated the whitening tendency of brown adipose tissue. Lipidomics analysis revealed that ABFM intake significantly improved abnormal hepatic lipid metabolic profiles, notably downregulating the expression levels of diacylglycerol (DG) and phosphatidylglycerol (PG), while markedly ameliorating sphingolipid metabolism disorders and ceramide (Cer) levels, which are highly associated with liver fibrosis. These findings further elucidate the mechanisms by which adzuki bean flavonoid components improve diet-induced obesity and associated liver injury, providing a theoretical basis for exploring safe and effective dietary intervention strategies based on plant flavonoids.

## 1. Introduction

The persistently rising prevalence of overweight and obesity, coupled with their strong associations with type 2 diabetes, cardiovascular diseases, psychological disorders, and even certain cancers, has gradually emerged as a major global public health challenge [1]. The primary driver of overweight and obesity is the energy imbalance caused by excessive intake and insufficient expenditure due to high-fat diets [2]. Current strategies for preventing and managing overweight and obesity include lifestyle (dietary) interventions, anti-obesity medications, and surgical interventions [3]. However, while dietary interventions yield short-term effects, much of the initial weight loss stems from glycogen and water depletion, with long-term weight loss slowing down [3,4]. Although pharmacological and surgical interventions show some efficacy, they face limitations such as insufficient effectiveness, safety concerns, and uncertain long-term benefits [4,5,6]. For instance, orlistat, a representative over-the-counter anti-obesity drug, may cause gastrointestinal irritation [7], hepatorenal toxicity [8], and impaired absorption of fat-soluble vitamins (A, D, E, and K) [9]. In contrast, a substantial body of experimentally validated research suggests that natural polyphenols, such as flavonoids, administered through food or nutritional fortification, may serve as safer and more effective alternatives for combating obesity [10,11].

As a class of abundant and widely distributed polyphenolic secondary metabolites in plant-based diets [12], flavonoids are characterized by a 2-phenylchromone (C6-C3-C6) tricyclic skeleton composed of 15 carbon atoms. They are further classified into various subclasses—such as flavonols, flavones, flavanones, flavan-3-ols, and isoflavones—based on different substituents, with over 9000 such compounds identified in nature [13]. Flavonoids exhibit diverse bioactivities, and their dietary intake can prevent or mitigate the onset of various chronic non-communicable diseases, including cardiovascular diseases, type 2 diabetes, cancer, neurodegenerative disorders, inflammation, and obesity [12,14]. Their anti-obesity properties are primarily mediated through appetite suppression, increased energy expenditure, gut microbiota modulation, and inhibition of adipogenesis [15]. For example, apigenin has been shown to ameliorate dyslipidemia and hepatic steatosis in HFD-fed mice by reducing the activity of hepatic enzymes involved in triglyceride and cholesterol esterification while downregulating genes associated with hepatic lipogenesis and lipolysis [16]. Studies on HFD-induced obese mice and HepG2 cells have demonstrated that Pueraria flavonoids activate autophagy via the PI3K/Akt/mTOR signaling pathway, thereby reducing intracellular lipid accumulation and inflammation and alleviating the pathological progression of non-alcoholic fatty liver disease (NAFLD) [17]. Additionally, the flavonol kaempferol has been reported to exert anti-obesity effects by targeting the hypothalamus, a key brain region regulating energy balance. Kaempferol mitigates obesity-related hypothalamic inflammation by reducing the release of pro-inflammatory cytokines (e.g., TNF-α, IL-1β) via pathways such as TLR4/NF-κB, thereby attenuating microglial overactivation [18].

Adzuki bean (*Vigna angularis*) is an annual leguminous plant native to East Asia and widely cultivated globally, representing one of the earliest domesticated crops [19]. Rich in bioactive compounds such as polyphenols, saponins, polysaccharides, and bioactive peptides [20], adzuki beans offer multiple health benefits, including antioxidant, anti-inflammatory, anti-obesity, anti-diabetic, antimicrobial, and neuroprotective effects [21,22]. Given their exceptional nutritional value and bioactivity, adzuki beans have garnered increasing attention as a nutritious and safe legume, serving as a potential raw material for chronic disease prevention and functional food development. Studies using adzuki bean saponins to intervene in HepG2 (lipid accumulation) and 3T3-L1 (adipogenesis) cell models revealed that these saponins activate the PI3K/Akt/GSK3β/β-catenin/c-Myc pathway, improving lipid metabolism and mitochondrial function in HepG2 hepatocytes while suppressing adipogenesis by modulating β-catenin signaling and downregulating C/EBPα and PPARγ [23]. Furthermore, in vitro molecular docking simulations demonstrated that certain peptides derived from heat-treated adzuki bean protein hydrolysates exhibit strong inhibitory effects on pancreatic lipase (PL) and cholesterol esterase (CE). These peptides occupy the catalytic sites (e.g., Ser153 and His264 in PL; Ser194 and His435 in CE) or substrate-binding sites (e.g., Ala108 in CE) of PL and CE via hydrogen bonds, hydrophobic interactions, salt bridges, and π-π stacking, thereby inhibiting enzyme activity. Notably, these target peptides are non-toxic, non-carcinogenic, and exhibit potential multifunctionality (e.g., anti-inflammatory, antihypertensive) [24]. Additionally, although there are no reports on weight loss efficacy, the bioactivity of multi-component flavonoid pure compound mimics has been demonstrated to be comparable to that of natural extracts and comprehensively superior to single-component flavonoids [25]. As a legume with high flavonoid content [26], adzuki bean flavonoids also demonstrate anti-obesity effects [27]. However, the composition of natural extracts is complex, the active ingredients responsible for their effects are unclear, and their mechanisms of action—particularly from the perspectives of histopathology and lipidomics—remain unclear. Therefore, this study employed LC-MS techniques to identify the flavonoid composition in adzuki beans. Based on the identification results, a high-purity adzuki bean flavonoid mimic (ABFM) was reconstituted using pure flavonoids in proportional ratios. The weight-loss and lipid-lowering effects of ABFM were evaluated in HFD-induced obese mice, and the mechanisms were elucidated through histopathological analysis of adipose tissue and liver, as well as hepatic lipidomics. This research provides a theoretical foundation for developing functional foods based on adzuki bean flavonoids and further exploring safe and effective anti-obesity and lipid-lowering bioactive components in plant flavonoids.

## 2. Materials and Methods

### 2.1. Materials and Key Reagents

Red adzuki beans were purchased from the local market, with the variety being zhenzhuhong. High-fat animal feed was procured from Changchun Yisi Laboratory Animal Technology Co., Ltd. (Changchun, China); the standard animal feed was obtained from Beijing Ke’ao Xieli Feed Co., Ltd. (Beijing, China); orlistat (active control drug) was purchased from Shanghai Yuanye Bio-Technology Co., Ltd. (Shanghai, China); and the flavonoid standards (daidzein, genistin, genistein, rutin, daidzin, apigenin, astragalin, and naringenin, purity ≥ 97%) used for configuring ABFM were sourced from Shanghai Aladdin Biochemical Technology Co., Ltd. (Shanghai, China). The quantitative assay reagent kits for serum indices such as total triglycerides (to distinguish it from “triglyceride-TG” in the lipidomics section, serum total triglycerides is abbreviated as tTG), alanine aminotransferase (ALT), and aspartate aminotransferase (AST) were purchased from Chengdu Seamaty Science & Technology Co., Ltd. (Chengdu, China); the total cholesterol (TC) assay kit, as well as the high-density lipoprotein (HDL-c) and low-density lipoprotein (LDL-c) assay kits, were acquired from Beijing Solarbio Science & Technology Co., Ltd. (Beijing, China).

### 2.2. Analysis of Flavonoid Composition in Adzuki Beans

#### 2.2.1. Sample Extraction

After cleaning, the red adzuki beans were soaked to achieve full expansion and softening. An appropriate amount was then taken for flavonoid extraction using methanol. The mixture was vortexed and subsequently placed in a tissue grinder, where it was subjected to vortex grinding at least twice. Following ultrasonication at room temperature for 15 min and centrifugation at 12,000 rpm and 4 °C for 5 min, the supernatant was collected and filtered through a 0.22 μm membrane filter, ready for instrumental analysis.

#### 2.2.2. LC-MS Analysis

The chromatographic separation was performed on an ACQUITY Ultra Performance Liquid Chromatography (UPLC) system (Waters Corporation, Milford, MA, USA). Mass spectrometric detection of flavonoids was conducted using an AB5000 mass spectrometer (AB SCIEX, Boston, MA, USA) equipped with an electrospray ionization (ESI) source, employing multiple reaction monitoring (MRM) for scanning. UPLC separation was carried out on an ACQUITY UPLC BEH C18 column (2.1 × 100 mm, 1.7 μm). The injection volume was 5 μL, with gradient elution at a flow rate of 0.25 mL/min using 0.1% formic acid in water (mobile phase A) and methanol (mobile phase B) under the following conditions: 0–1 min, 10% B; 1–3 min, 10–33% B; 3–10 min, 33% B; 10–15 min, 33–50% B; 15–20 min, 50–90% B; 20–21 min, 90% B; 21–22 min, 90–10% B; 22–25 min, 10% B.

The ESI-MRM/MS analytical parameters were set as follows: negative ionization mode, ion source temperature 500 °C, ion source voltage −4500 V, collision gas 6 psi, and both nebulizer and auxiliary gas at 50 psi.

### 2.3. Animal Experiment Design

#### 2.3.1. Preparation of Red Adzuki Beans Flavonoid Mimic (ABFM)

Based on the LC-MS analysis results of the flavonoid composition in red adzuki beans, the top eight flavonoid compounds (accounting for 98.66% of the total content) were selected. After content conversion, an ABFM was formulated with the following composition: daidzein (33.19%), genistin (23.65%), genistein (22.33%), rutin (10.56%), daidzin (5.42%), apigenin (2.24%), astragalin (1.36%), and naringenin (1.25%). During the intervention period, the mixture was prepared and aliquoted weekly, stored at −20 °C, and thawed at 4 °C for one day before use, gradually returning to room temperature prior to application.

#### 2.3.2. Animal Experiment Design and Sample Collection

All experimental procedures and treatments were approved by the Animal Experiment Ethics Committee of Heilongjiang Bayi Agricultural University (Approval No. SPXY2023015) and strictly adhered to the “Regulations for the Administration of Affairs Concerning Experimental Animals”. Six-week-old male C57BL/6 mice, specific pathogen-free (SPF) grade, with a body weight of 20 ± 2 g, were purchased from the Experimental Animal Department of Harbin Medical University (License No. SCXK(Hei)2019-001). The housing environment was maintained at a temperature of 23 ± 2 °C, relative humidity of 50 ± 5%, with a 12 h light/dark cycle. After one week of acclimatization feeding, the mice were randomly divided into two groups based on body weight. The model group was gradually transitioned to a high-fat diet (HFD) using a 7-day dietary switch method, while the normal group continued to receive a normal-fat diet (NFD). After 5 weeks of modeling, the model group exhibited an average body weight exceeding that of the normal group by 20%, indicating successful model establishment. Subsequently, a 6-week gavage intervention was initiated. The specific gavage protocols were as follows: Normal group (N): normal diet + 0.5% sodium carboxymethyl cellulose (CMC-Na) suspension. Model control group (M): high-fat diet + 0.5% CMC-Na suspension. Active control drug group (AC): high-fat diet + 25 mg/kg BW orlistat. High-dose intervention group (IG-H): high-fat diet + 200 mg/kg BW flavonoids. Medium-dose intervention group (IG-M): high-fat diet + 100 mg/kg BW flavonoids. Low-dose intervention group (IG-L): high-fat diet + 50 mg/kg BW flavonoids. Orlistat or ABFM were uniformly mixed into a 0.5% CMC-Na suspension.

At the end of the intervention, after a 12 h fasting period (with water provided ad libitum), body length was measured, and the mice were euthanized via cervical dislocation. Blood samples were collected and centrifuged at 10,000 rpm at 4 °C to obtain serum for biochemical analysis. Additionally, tissues including the liver, kidneys, epididymal fat, and interscapular brown adipose tissue were harvested, cleaned, and weighed. Samples of liver and adipose tissues were fixed in 4% paraformaldehyde and subsequently subjected to histopathological examination via hematoxylin–eosin (H&E) staining, Oil Red O staining, and Masson staining. Furthermore, portions of liver samples were flash-frozen in liquid nitrogen and stored at −80 °C for lipid metabolomics analysis.

### 2.4. Biochemical Parameters and Histological Analysis

The concentrations of serum tTG, TC, AST, ALS, HDL-c, and LDL-c were measured using quantitative detection reagent discs and serum biochemical reagent kits in accordance with the manufacturer’s instructions.

For histomorphological observation, both paraffin and frozen tissue sections were prepared. Paraffin sections were produced by fixing the tissues, dehydrating them through a graded ethanol series, clearing, infiltrating with wax, embedding, and then cutting into 2–5 μm thin sections using a paraffin microtome for subsequent H&E and Masson staining. Frozen sections were prepared by embedding sucrose-gradient-dehydrated tissue blocks in OCT compound, rapidly freezing them, and cutting into 8–10 μm thick sections, primarily for Oil Red O fat staining. Regarding staining procedures, conventional pathological structure observation was performed using H&E staining; collagen fiber assessment was conducted using Masson’s trichrome staining; and lipid deposition was visualized via Oil Red O staining with hematoxylin counterstaining for nuclei. After staining, paraffin sections were dehydrated, cleared with xylene, and mounted with neutral resin, while frozen sections were directly mounted with glycerin gelatin. All prepared sections were stored away from light for future use. The results of H&E staining and Masson staining were subjected to panoramic scanning of the sections using the 3DHISTECH digital pathology scanning system, and image acquisition and cell size measurement were performed using SlideViewer 2.9 software. The results of Oil Red O staining were microscopically photographed, and the stained lipid droplets were identified and counted using Media Cybernetics Image-Pro Plus 6.0 software.

### 2.5. Hepatic Lipid Metabolomics Analysis

#### 2.5.1. Liver Sample Processing and Metabolite Extraction

Total lipids were extracted from the liver tissues of mice in the normal group (N Group), model control group (M Group), and low-dose intervention group (IG Group). For each 50 mg sample, 280 μL of extraction solution (methanol/water = 2:5) was added, followed by 400 μL of MTBE. The samples were cryogenically ground at −10 °C and 50 Hz, then subjected to low-temperature ultrasonic extraction for 30 min, followed by incubation at −20 °C for 30 min. After centrifugation at 13,000× *g* and 4 °C, 350 μL of the supernatant was collected and dried under nitrogen gas. The residue was reconstituted in 100 μL of reconstitution solution (isopropanol/acetonitrile = 1:1). The mixture was vortexed for 30 s and sonicated in an ice-water bath at 40 kHz for 5 min. Following high-speed centrifugation at 13,000× *g* and 4 °C for 10 min, the supernatant was collected for subsequent analysis.

#### 2.5.2. LC-MS/MS Analysis

LC-MS/MS analysis of lipids was performed on an ultra-high-performance liquid chromatography coupled with Fourier transform mass spectrometry (UHPLC-Q Exactive HF-X) system (Thermo Fisher Scientific, Waltham, MA, USA). UHPLC separation was conducted using an Accucore C30 column (100 mm × 2.1 mm, 2.6 μm) with an injection volume of 2 μL. The mobile phases consisted of solvent A (10 mM ammonium acetate in 50% acetonitrile aqueous solution containing 0.1% formic acid) and solvent B (2 mM ammonium acetate in acetonitrile/isopropanol/water (10/88/2) containing 0.02% formic acid), with a flow rate of 0.40 mL/min. The gradient elution program was as follows: 0–4 min, 35–60% B; 4–12 min, 60–85% B; 12–15 min, 85–100% B; 15–17 min, 100% B; 17–18 min, 100–35% B; 18–20 min, 35% B. Mass spectrometric data acquisition was performed in both positive and negative ion modes, with a scan range of m/z 200–2000. The ion spray voltages were set at ±3000 V for positive and negative modes, respectively, with sheath gas at 60 psi, auxiliary heating gas at 20 psi, ion source heating temperature at 370 °C, and collision energy cycling at 20-40-60 V. The raw LC-MS/MS data from lipidomics were imported into the lipidomics processing software LipidSearch (Thermo Fisher Scientific) for baseline filtering, peak identification, integration, retention time correction, peak alignment, and identification, ultimately generating a data matrix containing lipid names, retention times, mass-to-charge ratios, and peak intensities.

### 2.6. Data Processing

The physiological and biochemical index data obtained in this study are presented as the mean ± standard deviation (SD), and variability analysis was performed using IBM SPSS Statistics 27 software. The normality of the data was assessed using the Shapiro–Wilk test. Data that met the assumptions of normal distribution and homogeneity of variances were analyzed using one-way ANOVA, with post hoc Tukey HSD multiple comparison tests for between-group comparisons. The matrix data obtained from lipidomics database searching were uploaded to the Majorbio Cloud Platform (https://cloud.majorbio.com) for data analysis. Differential metabolites among two groups were summarized and mapped onto their biochemical pathways through metabolic enrichment and pathway analysis based on KEGG (kegg_v20221012) database search; scipy.stats (Python packages) (https://docs.scipy.org/doc/scipy/, accessed on 25 March, 2021 ) was exploited to identify statistically significantly enriched pathway using Fisher’s exact test. Statistical significance of all results is denoted by “*”: ns, not significant; *, *p* < 0.05; **, *p* < 0.01; ***, *p* < 0.001; ****, *p* < 0.0001.

## 3. Results

### 3.1. Flavonoid Composition in Adzuki Beans

The flavonoids in red adzuki beans were determined using an LC-MS system. As shown in Table 1, by comparison with standard compounds, it was found that the flavonoids in red adzuki beans primarily consist of isoflavones such as daidzein, genistin, genistein, and daidzin; flavonols such as rutin, astragalin, isoquercitrin, and kaempferol; flavones such as apigenin, vitexin, and isovitexin; flavanones such as naringenin and liquiritigenin; and dihydroflavonols such as dihydroquercetin. Among these, isoflavones accounted for the highest proportion (83.46%). The detailed flavonoid composition and their respective proportions are presented in Table 1.

### 3.2. Effects of ABFM Intake on Body Weight and Organ Indices

**Figure 1:**
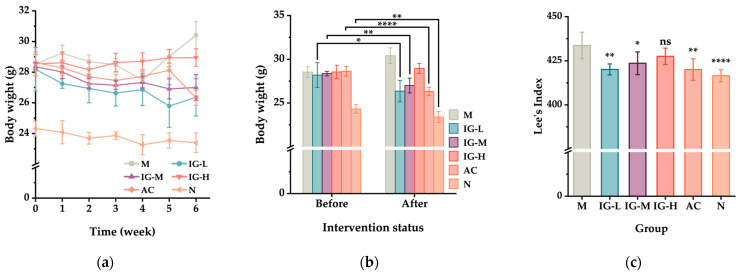

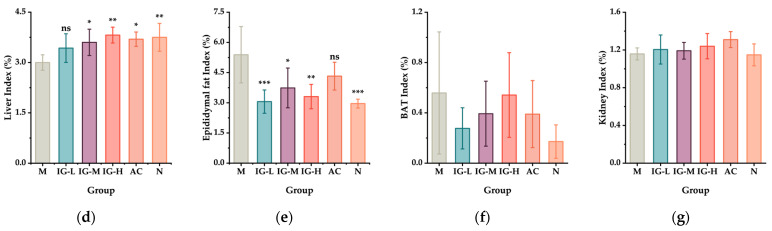
Effects of ABFM supplementation on body weight, Lee’s index, and organ indices in mice. (**a**) Changes in body weight during the intervention period; (**b**) comparison of body weight before and after the intervention; (**c**) Lee’s index; (**d**) liver index; (**e**) epididymal fat index; (**f**) BAT index; (**g**) kidney index. Data are presented as the mean ± standard deviation (*n* = 6). In the figure: IG-L represents the low-dose intervention group, IG-M represents the medium-dose intervention group, IG-H represents the high-dose intervention group, AC represents the active control drug group, M represents the model control group, and N represents the normal group; “*” indicates the level of statistical significance compared to the M group: ns, not significant; *, *p* < 0.05; **, *p* < 0.01; ***, *p* < 0.001; ****, *p* < 0.0001.

After 5 weeks of modeling feeding, there were no significant differences in initial body weight among the high-fat diet (HFD)-fed model groups at the end of the modeling period. However, the body weight and Lee’s index of the model groups were significantly higher than those of the normal diet (NFD)-fed control group. During the first 3 weeks of intervention, changes in body mass were minimal, but from the fourth week onward, significant variations were observed across all groups (Figure 1a). Within the model groups, the AC group, which was administered the positive control drug orlistat, exhibited the most pronounced weight loss, with a reduction of 2.31 g (8.76%). In the IG group treated with ABFM, low- and medium-dose ABFM interventions resulted in significant weight loss, with reductions of 1.83 g (6.94%) and 1.36 g (5.04%), respectively, whereas the high-dose group showed no significant weight loss (Figure 1b). Concurrently, the M group displayed a highly significant increase in body mass (*p* < 0.01), with a gain of 6.56%. These findings indicate that ABFM intervention within a certain dosage range significantly inhibits or reduces body mass gain in diet-induced obese mice under high-fat diet conditions. As shown in Figure 1c, Lee’s index across the groups reflected a similar trend, wherein low- and medium-dose interventions were more effective than high-dose interventions.

Figure 1 and Table 2 illustrate the effects of ABFM supplementation on the weights and organ indices of the liver, epididymal fat, interscapular brown adipose tissue, and kidneys. As depicted in Figure 1d, compared to the N group, 11 weeks of HFD feeding significantly increased both the mass and organ index of epididymal fat in the M group. In contrast, FABS intervention markedly reduced the mass and organ index of epididymal fat in mice. Relative to the M group, the IG-L group exhibited a 98.70% reduction in epididymal fat mass, the IG-M group a 54.55% reduction, and the IG-H group a 64.52% reduction. Notably, the IG-L group, which demonstrated the most significant weight loss, also had significantly lower epididymal fat mass than the AC group. Regarding brown adipose tissue (Figure 1e), the organ mass of brown fat was positively correlated with the dosage of ABFM, suggesting that ABFM promotes the generation of brown adipose tissue and enhances energy expenditure in mice. In terms of the liver, the increased body mass and fat accumulation in the M group led to a significantly lower liver organ index compared to the N group. However, the IG group treated with flavonoids and the AC group treated with orlistat showed no significant differences in liver organ index compared to the N group.

### 3.3. Effects of ABFM Intake on Serum Biochemical Indices

**Figure 2:**
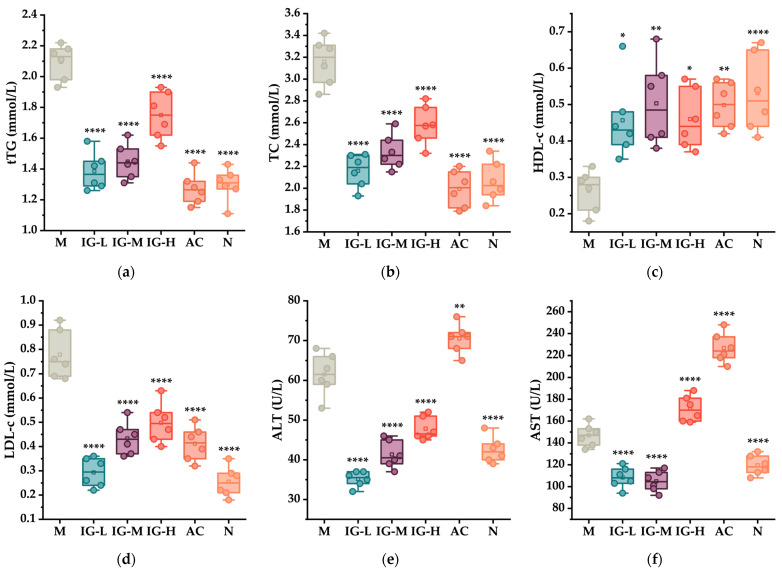
The effects of ABFM supplementation on serum lipid-related and liver function-related indicators in mice (*n* = 6). (**a**) Impact on total triglycerides (to distinguish it from “triglyceride-TG” in the lipidomics section, serum total triglycerides is abbreviated as tTG). (**b**) Impact on total cholesterol (TC). (**c**) Impact on high-density lipoprotein (HDL-c). (**d**) Impact on low-density lipoprotein (LDL-c). (**e**) Impact on alanine aminotransferase (ALT). (**f**) Impact on aspartate aminotransferase (AST). “*” indicates the statistical significance compared to the M group: *, *p* < 0.05; **, *p* < 0.01; ****, *p* < 0.0001.

Obesity induced by a high-fat diet (HFD) is often accompanied by abnormal lipid metabolism, characterized by dyslipidemia [28]. As shown in Figure 2a–d, compared to the normal diet group (N group), mice in the HFD-fed group (M group) exhibited significantly elevated serum levels of total triglycerides (to distinguish it from “triglyceride-TG” in the lipidomics section, serum total triglycerides is abbreviated as tTG), total cholesterol (TC), and low-density lipoprotein (LDL-c), along with a significant decrease in high-density lipoprotein (HDL-c) levels (*p* < 0.05). However, intervention with ABFM effectively reversed this phenomenon, significantly ameliorating the dyslipidemia in serum. Among the three intervention dose groups, the efficacy was inversely proportional to the dose, with the low-dose group (50 mg/kg/d BW) demonstrating the most pronounced effects. Regarding the serum liver function markers alanine aminotransferase (ALT) and aspartate aminotransferase (AST), which serve as indicators of hepatocyte health, HFD feeding significantly increased both markers (*p* < 0.05), suggesting that prolonged HFD consumption may have induced hepatocyte damage. ABFM intake effectively alleviated HFD-induced liver dysfunction, with the low-dose intervention group showing the most significant improvement, similar to the lipid profile results (Figure 2e,f). Additionally, the active control group (AC) exhibited a substantial increase in ALT and AST levels, indicating that prolonged orlistat intake may adversely affect hepatocytes.

### 3.4. Effects of ABFM Intake on Histological Degeneration

#### 3.4.1. H&E Staining of Adipose Tissue

**Figure 3:**
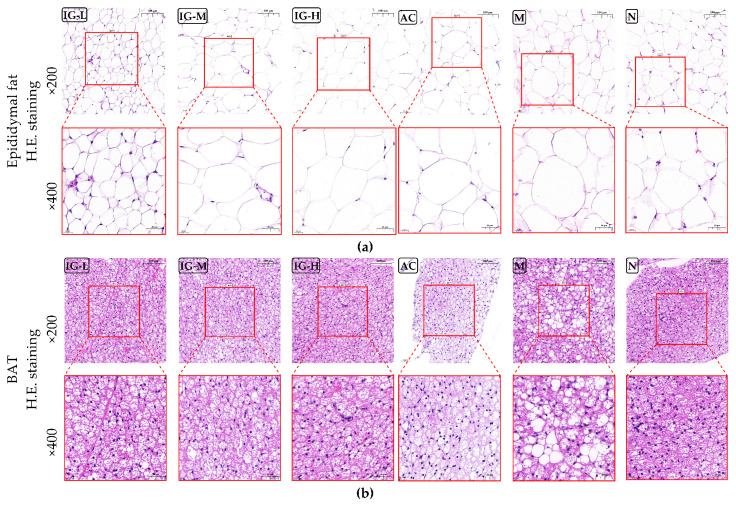
Histopathological images of H&E staining in epididymal fat (**a**) and BAT (**b**). (Magnification: ×200 and ×400.)

After 6 weeks of intervention with ABFM and orlistat, as shown in Figure 3a, the IG-L group exhibited the most significant intervention effect. The fat droplets in the epididymal adipose tissue were smaller in volume, more rounded in shape, and the tissue appeared more compact. The fat droplets in the other groups were similar to those in the N group, with relatively uniform particle sizes, no giant cell bodies observed in the entire field of view, intact cellular structures, and clearly visible nuclei and cytoplasm. In contrast, the epididymal adipose tissue of the M group mice contained larger fat droplets compared to the other groups, with the presence of large vacuolated fat chambers, resulting in the nuclei and organelles of the adipocytes being compressed and difficult to observe in the field of view. Additionally, the adipocytes varied in size.

The results of H&E staining of the interscapular brown adipose tissue in mice are shown in Figure 3b. The tissue morphology of the ABFM intervention group was similar to that of the N group, displaying small, nearly circular fat chambers surrounded by abundant capillary structures. Although the AC group had a similar brown adipocyte structure, it was sparser and more loosely arranged. In contrast, the brown adipocyte structure of the M group differed from the other groups, with larger cell volumes and the presence of large, single vacuolated fat chambers, indicating a trend toward whitening of the brown adipose tissue [29].

#### 3.4.2. Oil Red O Staining of Liver

**Figure 4:**
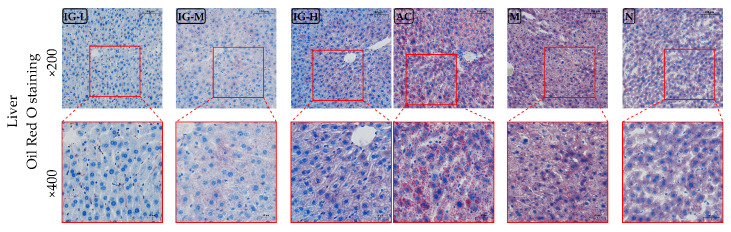
Histopathological images of liver tissue stained with Oil Red O. (Magnification: ×200 and ×400.)

The results of Oil Red O staining for mouse liver are shown in Figure 4. The findings indicate that the M group exhibited severe hepatic lipid deposition, with a total of 1955 ± 98 lipid droplets identified at 400× magnification. Lipid droplets were widely and uniformly distributed among hepatocytes. In contrast, the IG group (IG-L: 0 lipid droplets, IG-M: 279 ± 28 lipid droplets, IG-H: 94 ± 54 lipid droplets) and N groups (1054 ± 49 lipid droplets) displayed fewer lipid droplets in the liver structure, which were also relatively smaller in size, suggesting that ABFM significantly reduces hepatic fat deposition. Although the number of lipid particles decreased in the orlistat intervention group (1353 ± 104 lipid droplets), their distribution was uneven and diffuse, implying that while orlistat exerts weight-reducing and lipid-lowering effects, it may concurrently induce hepatic injury.

#### 3.4.3. Masson’s Staining of Liver

**Figure 5:**
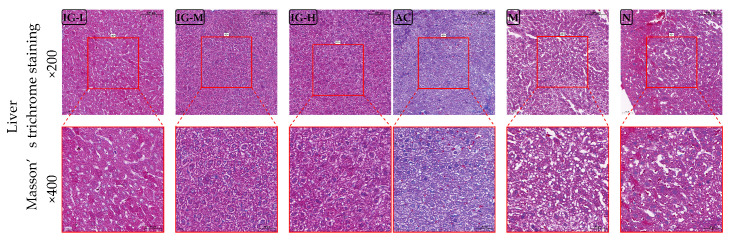
Histopathological images of liver tissue stained with Masson’s trichrome. (Magnification: ×200 and ×400.)

The Masson staining results of mouse liver tissues are shown in Figure 5. The degree of liver fibrosis was reduced in the N, IG, and M groups, with tightly distributed hepatocytes and no observable signs of liver fibrosis within the field of view. In contrast, the AC group exhibited extensive fibrosis under the influence of orlistat, with widespread fibrotic tissue distribution. This indicates that ABFM does not cause liver damage while achieving the expected weight loss and lipid-lowering effects, whereas orlistat may induce a certain degree of liver injury.

### 3.5. Effects of ABFM Intake on Lipid Metabolomics

#### 3.5.1. High-Fat Diet-Induced Hepatic Lipid Metabolism Disorder in Mice

**Figure 6:**
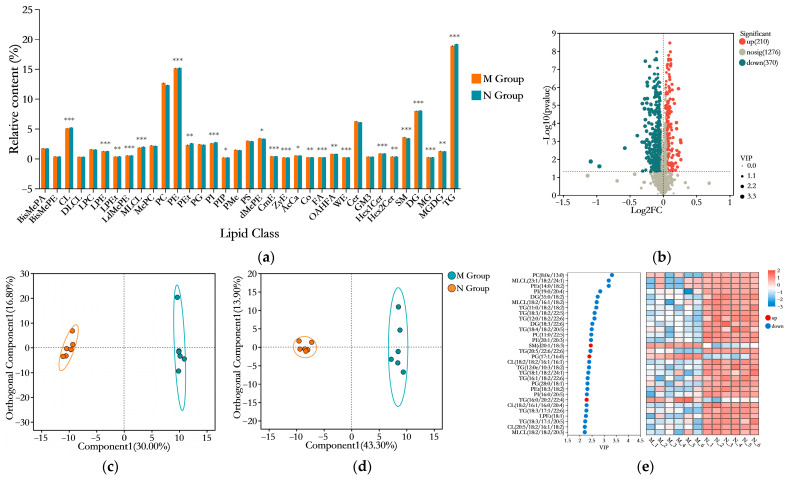
Effects of HFD feeding on hepatic lipid metabolism. (**a**) The bar chart illustrates the differences in lipid species and their relative content in the liver of mice following HFD treatment (*n* = 6). “*” indicates the statistical significance compared to the M group: *, *p* < 0.05; **, *p* < 0.01; ***, *p* < 0.001. (**b**) The volcano plot displays the differential expression of lipids in the liver between the M and N groups of mice. (**c**,**d**) Orthogonal partial least squares–discriminant analysis (OPLS-DA) models of liver lipids in the M and N groups under cation (**c**) and anion (**d**) modes. (**e**) The variable importance in projection (VIP) score plot of the OPLS-DA model for liver lipids in the M and N groups, highlighting the top 30 lipids with VIP > 1.

A high-fat diet (HFD) not only affects the physiological, biochemical, and histopathological aspects of obese mice but also influences the hepatic lipid metabolic profile. Therefore, we conducted a comparative analysis of the differential lipids in the livers of mice from the HFD-fed M group and the normal diet-fed N group. As shown in Figure 6b, a total of 580 differentially expressed lipid metabolites were identified (screening criteria: *p* < 0.05, VIP > 1, and (FC < 1 or FC > 1)), among which 210 were upregulated and 370 were downregulated compared to the N group. These included 108 triglycerides (TGs), 87 phosphatidylethanolamines (PEs), 70 phosphatidylcholines (PCs), 46 cardiolipins (CL/DLCL/MLCLs), 44 diglycerides (DGs), 35 ceramides (Cers), 21 dimethylphosphatidylethanolamines (dMePEs), 19 sphingomyelins (SMs), 18 phosphatidylserines (PSs), 18 phosphatidylinositols (PIs), 16 phosphatidylethanols (PEts), 14 phosphatidylglycerols (PGs), 12 methyl phosphatidylcholines (MePCs), 9 phosphatidylmethanols (PMes), 9 bis-methyl phosphatidic acids (BisMePAs), 8 Monogalactosyldiacylglycerols (MGDGs), 8 lysophosphatidylcholines (LPCs), 7 lysophosphatidylethanolamines (LPEs), 7 simple glc series (Hex1Cer/Hex2Cers), 5 OAcyl-(gamma-hydroxy)FA (OAHFAs), 3 lysodimethylphosphatidylethanolamines (LdMePEs), 3 acyl carnitines (AcCas), 2 bis-methyl phosphatidylethanolamines (BisMePEs), 2 campesterol esters (CmEs), 2 gangliosides (GM3s), 2 lyso-phosphatidylethanols (LPEts), 1 coenzyme (Co), 1 free fatty acid (FA), 1 monoglyceride (MG), 1 wax ester (WE), and 1 zymosterol ester (ZyE). As shown in Figure 6a, compared to the N group, high-fat diet (HFD) feeding significantly increased the levels of AcCa, LdMePE, MG, MGDG, SM, and dMePE in the M group. Conversely, the N group exhibited significantly higher levels of CL, CmE, Co, DG, FA, Hex1Cer, Hex2Cer, LPE, LPEt, MLCL, OAHFA, PE, PEt, PI, PIP, TG, WE, and ZyE compared to the M group (*p* < 0.05). To further investigate the impact of HFD on hepatic lipidomic profiles, multivariate statistical analysis was performed. An orthogonal partial least squares–discriminant analysis (OPLS-DA) model was constructed to visualize the data. As shown in Figure 6c,d, under both negative and positive ion modes, the hepatic lipid composition exhibited significant differences between the two feeding regimens, with clear separation. Figure 6e displays the top 30 lipid metabolites ranked by variable importance in projection (VIP) scores, among which TG constituted the largest proportion, followed by cardiolipins (CL/MLCL). The heatmap revealed that the expression of these 30 metabolites was predominantly downregulated, with only three metabolites upregulated: SM (d20:1/18:3), PG (17:1/16:0), and TG (16:0/20:2/22:4).

**Figure 7:**
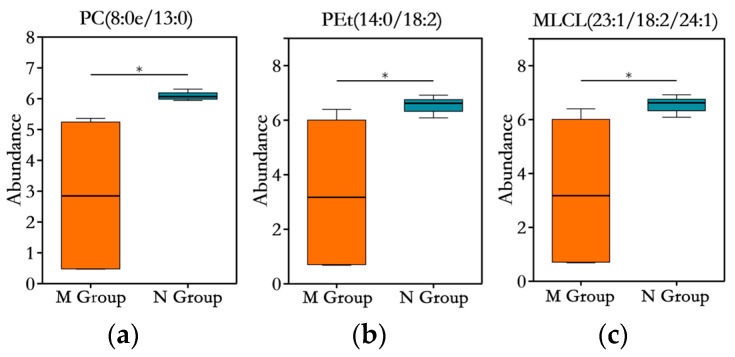
The relative expression levels of differential metabolites (*n* = 6) screened based on the criteria of VIP > 3 and *p* < 0.05 in the OPLS-DA model. “*” indicates the statistical significance compared to the N group: *, *p* < 0.05. (**a**) boxplot of abundance distribution for PC (8:0e/13:0); (**b**) PEt (14:0/18:2); (**c**) MLCL (23:1/18:2/24:1).

To further screen for differential lipids, the selection criteria were narrowed down to VIP > 3 in the OPLS-DA model and *p* < 0.05, resulting in the identification of three differential metabolic lipids, as illustrated in Figure 7. These lipids, all belonging to the glycerophospholipid (GP) category, include PC (8:0e/13:0), PEt (14:0/18:2), and MLCL (23:1/18:2/24:1). All three lipids were downregulated in the HFD model group.

#### 3.5.2. Effects of ABFM Supplementation on Hepatic Lipid Metabolism in Mice

**Figure 8:**
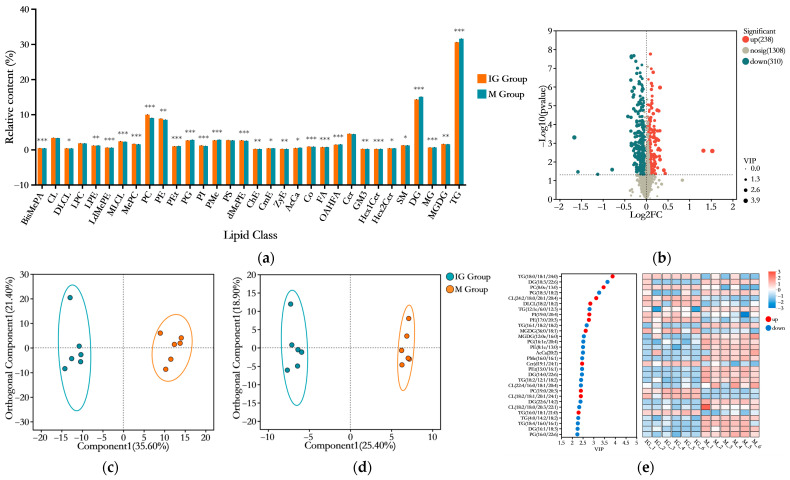
Effects of ABFM intervention on hepatic lipid metabolism. (**a**) The bar chart illustrates the differences in lipid species and relative content between the ABFM intervention group (IG group) and the non-intervention group (M group) (*n* = 6). (**b**) The volcano plot displays the differential expression of lipids in the liver of mice between the IG and M groups. The symbol “*” indicates statistical significance compared to the M group: *, *p* < 0.05; **, *p* < 0.01; ***, *p* < 0.001. (**c**,**d**) Orthogonal partial least squares–discriminant analysis (OPLS-DA) models of hepatic lipids in the IG and M groups under positive ion mode (**c**) and negative ion mode (**d**). (**e**) Variable importance in projection (VIP) score plot of the OPLS-DA model for hepatic lipids in the IG and M groups, highlighting the top 30 lipids with VIP > 1.

To investigate the effects of ABFM supplementation on hepatic lipid metabolism in HFD-induced obese mice, a comparative analysis of hepatic lipid metabolite composition was conducted between the HFD-fed M group and the ABFM-supplemented IG group. As illustrated in the volcano plot (Figure 8b), a total of 548 differential lipid metabolites were identified (screening criteria: *p* < 0.05, VIP > 1, and (FC < 1 or FC > 1)), among which 238 were upregulated and 310 were downregulated relative to the M group. These included 170 TGs, 77 DGs, 51 PCs, 47 PEs, 34 CLs/DLCLs/MLCLs, 24 Cers, 15 dMePEs, 15 PMes, 15 PGs, 15 PSs, 9 MGDGs, 9 OAHFAs, 9 LPCs, 8 MePCs, 7 PIs, 6 LPEs, 6 SMs, 5 PEts, 4 Cos, 3 AcCas, 3 FAs, 3 LdMePEs, 3 MGs, 3 Hex1Cers/Hex2Cers, 2 BisMePAs, 2 CmEs, 1 ChE, 1 GM3, and 1 ZyE. Figure 8a demonstrates that under ABFM intervention, lipids such as BisMePA, ChE, Co, DLCL, GM3, Hex2Cer, LdMePE, MGDG, MLCL, MePC, PC, PE, PI, SM, ZyE, and dMePE were significantly upregulated in the IG group, whereas AcCa, CmE, DG, FA, Hex1Cer, LPE, MG, OAHFA, PEt, PG, PMe, and TG were significantly downregulated (*p* < 0.05). To further elucidate the impact of ABFM intervention on hepatic lipid metabolic profiles, multivariate statistical analysis was performed by constructing an OPLS-DA model to visualize the lipidomics data from the two groups (Figure 8c,d). The results indicated clear separation between the groups in both positive and negative ion modes, suggesting that ABFM intake significantly influenced hepatic lipid metabolism. Figure 8e displays the top 30 differential lipids ranked by VIP scores between the two groups, among which 11 lipids were upregulated. These included glycerolipids (GLs) such as TG(18:0/18:1/24:0) and MGDG(36:0/18:1); glycerophospholipids (GPs) such as PC(8:0e/13:0), CL(24:2/18:0/20:1/20:4), DLCL(18:2/18:2), PI(19:0/20:4), PE(17:0/20:3), PC(19:0/20:3), and CL(18:2/18:1/20:1/24:1); and sphingolipids (SPs) such as Cer(d19:1/24:1) and TG(16:0/18:1/21:0).

**Figure 9:**
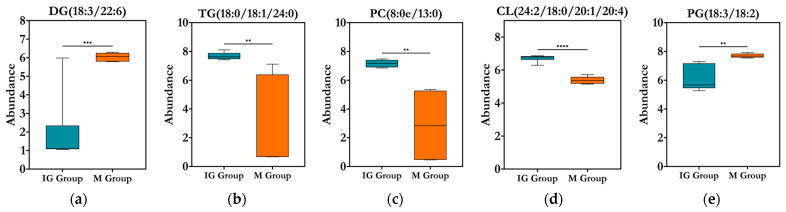
The relative expression levels of differential metabolites (*n* = 6) screened based on the criteria of VIP > 3 and *p* < 0.05 in the OPLS-DA model. “*” indicates the statistical significance compared to the M group: **, *p* < 0.01; ***, *p* < 0.001; ****, *p* < 0.0001. (**a**) boxplot of abundance distribution for DG(18:3/22:6); (**b**) TG(18:0/18:1/24:0); (**c**) PC(8:0e, 13:0); (**d**) CL(24:2/18:0/20:1/20:4); (**e**) PG(18:3/18:2).

Using the OPLS-DA model with VIP > 3 and *p* < 0.05 as criteria to narrow the screening scope, five differential metabolic lipids were identified (Figure 9). Among these, two were downregulated in the ABFM intervention group compared to the M group, namely DG(18:3/22:6) belonging to the GL category and PG(18:3/18:2) from the GP category. The remaining three were upregulated, including TG(18:0/18:1/24:0) from the GL category, as well as PC(8:0e, 13:0) and CL(24:2/18:0/20:1/20:4) from the GP category.

#### 3.5.3. KEGG Pathway Enrichment Analysis

**Figure 10:**
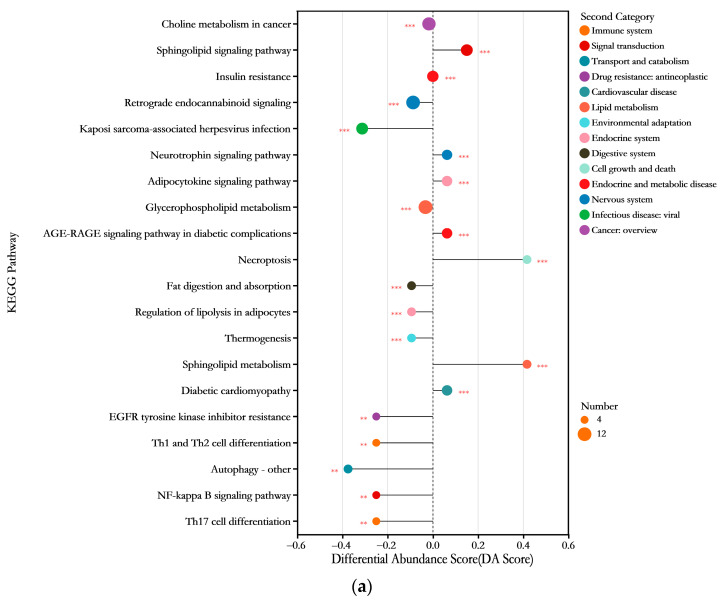

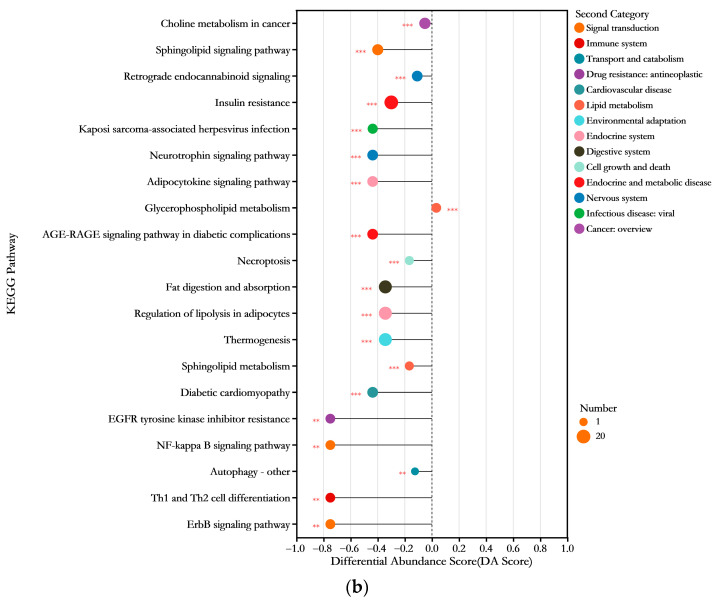
The KEGG metabolic pathway enrichment analysis based on significantly different lipid molecules between the M group vs. N group (**a**), and IG group vs. M group (**b**), displays the top 20 pathways ranked by *p*-value. The horizontal axis represents the Differential Abundance Score (DA score), which reflects the overall change in all metabolites of the metabolic pathway. A score of 1 indicates an upregulation trend in the expression of all annotated differential metabolites within the pathway, while a score of −1 indicates a downregulation trend. The length of the line segment corresponds to the absolute value of the DA score. The size of the dots represents the number of annotated differential metabolites in the pathway. “*” indicates the statistical significance compared to the M group: **, *p* < 0.01; ***, *p* < 0.001; .

By matching the metabolite information detected via UHPLC-MS to obtain KEGG compound IDs, the signaling pathways involving lipid metabolites in the liver tissues of HFD-induced obese mice were identified, thereby enabling an evaluation of potential functional lipid metabolism. As shown in Figure 10a, compared to the normal diet group (N group), the lipid metabolism pathways in the liver of HFD-fed mice exhibited significant downregulation (*p* < 0.05, DA score < 0) in pathways such as glycerophospholipid metabolism, fat digestion and absorption, regulation of adipocyte lipolysis, cholesterol metabolism, and glycerolipid metabolism. Conversely, pathways such as sphingolipid metabolism, linoleic acid metabolism, and α-linolenic acid metabolism were significantly upregulated. Under the intervention of ABFM, sphingolipid metabolism was significantly downregulated, while glycerophospholipid metabolism was significantly upregulated. Regarding other metabolic pathways, ABFM intervention also significantly promoted the downregulation of pathways such as insulin resistance, the AGE-RAGE signaling pathway in diabetic complications, and diabetic cardiomyopathy (Figure 10b).

## 4. Discussion

Polyphenolic compounds exert profound and multifaceted effects on lipid metabolism through a complex and interconnected set of molecular mechanisms. Plant-derived dietary polyphenols, due to their ability to ameliorate hepatic lipid metabolism disorders induced by high-fat diets (HFD) [30,31], demonstrate significant potential in weight management and combating obesity [32]. Although preliminary studies have confirmed the potential natural anti-obesity activity of flavonoids from red adzuki beans [27], the regulatory mechanisms underlying their effects on the pathological structure of adipose tissue and hepatic lipid metabolism profiles in HFD-induced obese mice remain insufficiently elucidated. This study systematically investigated the regulatory effects of red adzuki bean flavonoid components on hepatic lipid metabolism in HFD-induced obese mice using lipidomics technology. Lipids, the culprits of obesity, are also key molecules in maintaining cellular homeostasis, participating in ATP generation, vitamin/hormone synthesis, bile salt formation, cell membrane construction, and signal transduction. The distribution of lipids (e.g., abdominal fat) is closely associated with the risk of cancer and cardiovascular diseases [33]. As the central organ of lipid metabolism, the liver regulates systemic lipid balance through mechanisms such as the synthesis and secretion of lipoproteins, lipid uptake and storage, and interactions with peripheral tissues (e.g., white adipose tissue, brown adipose tissue) [34].

The results of this study indicate that, within a certain dosage range, ABFM intervention significantly suppresses body weight gain in diet-induced obese mice and markedly reduces the mass and index of epididymal adipose tissue. Serum biochemical analyses revealed that ABFM lowers serum levels of tTG (to distinguish it from “triglyceride-TG” in the lipidomics section, serum total triglycerides is abbreviated as tTG) and TC while improving HDL-c and LDL-c levels. Additionally, pathological sections demonstrated that ABFM inhibits the whitening transformation of brown adipose tissue, maintains its brown adipocyte morphology, ameliorates hepatic lipid droplet accumulation, and avoids the potential liver fibrosis damage caused by orlistat. Wang et al. reported similar findings, where intervention with a flavonoid-rich extract of *Millettia speciosa* in HFD-fed C57BL/6J obese mice significantly reduced body weight, liver mass, white adipose tissue, and blood glucose levels, while also lowering serum tTG, ALT, AST, and related inflammatory factors. Furthermore, the extract promoted thermogenesis in brown adipose tissue and activated lipolysis, fatty acid oxidation, and oxidative phosphorylation in white adipose tissue [35]. Burke et al. observed comparable but slightly different results, noting that citrus flavonoid intake reversed diet-induced obesity in Ldlr-/- mice by increasing energy expenditure and hepatic fatty acid oxidation, improving adipocyte size and number, insulin sensitivity, and hepatic steatosis. However, unlike our study, they did not observe evidence of white adipose tissue browning [36]. In contrast, Jung et al. found that low-dose apigenin intervention (0.005% apigenin in HFD) for 16 weeks in diet-induced obese C57BL/6J mice did not reduce body weight but improved lipid metabolism-related indicators such as plasma free fatty acids, TC, apolipoprotein B, and markers of liver dysfunction. Apigenin also reduced plasma pro-inflammatory mediators and fasting blood glucose levels [16]. These discrepancies may arise from differences in flavonoid sources and types. Peng et al. corroborated this view by studying the effects of two flavonoid monomers (rutin and quercetin) and flavonoid-rich tartary buckwheat on lipid metabolism in HFD-fed rats. Their results showed that the flavonoid monomers reduced body weight and serum levels of tTG, TC, LDL-c, TNF-α, and insulin while improving glucose tolerance, whereas tartary buckwheat intake further increased body weight. Nonetheless, all three interventions improved serum lipid profiles and reduced hepatic fat deposition, which is beneficial for preventing NAFLD [37]. These findings suggest that future research on the weight-reducing and lipid-lowering effects of flavonoids should consider the holistic impact of dietary intake on animal lipid metabolism, as whole-food consumption may yield different outcomes compared to isolated component intake. In addition to causing dyslipidemia, HFD-induced obesity can lead to varying degrees of liver injury, including elevated serum ALT and AST levels, hepatic fat deposition, hepatocyte vacuolization, sinusoidal dilation, and inflammatory cell infiltration—pathological features of non-alcoholic fatty liver disease (NAFLD) [38]. Flavonoids from *Rosa davurica* Pall. (RDPF) improved obesity and associated liver injury through multiple targets. Histopathological analysis revealed that RDPF alleviated hepatic fat deposition and hepatocyte vacuolization (steatosis) while improving hepatocyte morphology. It also reduced oxidative stress, enhanced antioxidant enzyme activity (SOD, CAT), lowered MDA levels, and modulated the expression of lipid metabolism-related genes, downregulating C/EBPα, SREBP-1C, and FAS while upregulating PPARα, ACOX1, and CAT [39]. It is noteworthy that the findings of this study indicate that the anti-obesity effects of ABFM were significant at low (50 mg/kg) and medium (100 mg/kg) doses but not at the high dose (200 mg/kg). This suggests that the anti-obesity effect of ABFM may not follow a linear dose–response relationship and could even exhibit effect saturation or inverse regulation. Green tea, due to its polyphenol content (particularly catechins), has potential anti-obesity properties. A dose–response meta-analysis of green tea supplements on human obesity markers revealed a non-linear relationship between green tea intake and weight loss effects. Within the supplementation range of 0–2000 mg/d, doses below 500 mg/d achieved the best weight loss outcomes, while increasing the dose further (500–1000 mg/d) resulted in diminished effects, with saturation observed beyond 1000 mg/d [40]. Moderate polyphenol intake can reduce adipogenesis, promote fatty acid oxidation, and increase energy expenditure by activating pathways such as AMPK/SIRT1 and inhibiting PPARγ, thereby exerting anti-obesity effects. However, high doses of polyphenols may induce prooxidant and pro-inflammatory effects, such as phenoloxy radical-mediated oxidative stress [41]. Moreover, the emergence of such a nonlinear dose–response relationship may also be related to bioavailability. Polyphenols exhibit low oral bioavailability and are rapidly metabolized by tissue enzymes (particularly liver enzymes) and gut microbiota in the human body, resulting in extremely low concentrations of free polyphenols in circulation. The doses of polyphenols used in clinical studies are generally much lower than those in in vitro experiments (μmol/L to mmol/L), while the plasma metabolite concentrations in humans rarely exceed nmol/L levels following normal dietary intake [41]. Additionally, a meta-analysis on the relationship between flavonoid intake and reduced risk of type II diabetes also demonstrated a non-linear dose–response pattern [42]. Interestingly, Snijman et al. investigated the dose–response effects of major flavonoids from *Aspalathus linearis* in interaction with two mutagens (2-AAF and AFB1) in Salmonella mutagenicity tests. The results revealed diverse dose–response relationships and mutagen-specific interactions. For instance, (-)-EGCG and quercetin exhibited typical concentration-dependent inhibitory or enhancing effects on both mutagens, while rutin and (+)-catechin showed biphasic effects, providing stronger protection against AFB1 at both high and low concentrations but weaker protection at intermediate concentrations [43]. This may be related to the different polyphenol structures. Research by Erk et al. on the dose–absorption relationship of various polyphenols in coffee (primarily chlorogenic acid compounds) revealed that the absorption mechanism of coffee polyphenols is passive diffusion. The absorption process is nonsaturable, and the absorption flux exhibits a linear relationship with the dose (R^2^ = 0.99). However, the absorption rate is independent of the dose, while physicochemical properties such as molecular size and lipophilicity influence absorption efficiency [44]. A study on the dose–response relationship of red wine pomace (rWPP) and white wine pomace (wWPP), both rich in polyphenols, revealed that rWPP did not exhibit a significant dose–response effect. The concentrations of total phenolic acid metabolites in plasma and urine did not consistently increase with higher intake doses (50, 100, 150, and 300 mg/kg BW). In contrast, wWPP demonstrated a linear dose–response relationship, particularly with significant increases at doses of 150 and 300 mg/kg BW. This difference may be attributed to the distinct polyphenol compositions of the two pomaces—such as the high anthocyanin content in rWPP and the abundance of flavanols and flavonols in wWPP—as well as their respective metabolic pathways [45]. The above report may suggest that the bioactivity of flavonoids (polyphenols) does not necessarily increase linearly with dosage. Excessive intake could potentially lead to adverse effects. The dose–response relationship and bioavailability may be associated with the composition and structure of flavonoids. It also indicates that future related research should place greater emphasis on the dosage, bioavailability, and safety of polyphenols.

Additionally, the hepatic lipid metabolomics results in this study demonstrated that ABFM intervention downregulated the expression of diglycerides (DGs) and phosphatidylglycerol (PG) in mouse livers, suppressed the expression of lipid metabolism-related functional pathways, and also reduced the expression of DG and simple glc series (HexCer) downstream of long-chain fatty acyl-CoA (LCFA-CoA) in the hepatic fatty acid biosynthesis pathway. This aligns with the findings reported by Feng et al., whose results indicated that nobiletin, a flavonoid, not only significantly inhibited body weight gain in HFD-induced obese rats and improved serum levels of tTG, TC, and LDL-c but also markedly ameliorated hepatic steatosis. Concurrently, lipid metabolomics revealed that these beneficial effects were associated with decreased levels of fatty acids, DG, TG, ceramide (Cer), and ST in the liver [46]. Furthermore, by integrating network pharmacology, lipidomics, and transcriptomics analyses, Du et al. investigated the mechanism by which flavonoids from vine tea (TF) ameliorate AKT-induced NAFLD. The results demonstrated that TF inhibits de novo fatty acid synthesis and modulates glycerophospholipid metabolism through the PPAR signaling pathway. Specifically, TF downregulates genes such as PPARγ and SCD1 to reduce fatty acid synthesis and regulates key genes including Dgkz, Chpt1, and Pla2g12b, thereby influencing glycerophospholipid metabolism. Concurrently, it reduces the accumulation of lipids such as TG, DG, PC, and PE [47]. By integrating lipidomics and proteomics, the mechanism underlying the preventive effects of flavonoids from *Cyclocarya paliurus* (CPF) on non-alcoholic steatohepatitis (NASH) was investigated. The results showed that CPF intake significantly reduced body weight, liver weight, tTG, and TC levels, as well as hepatic inflammation scores and fibrosis area, thereby improving hepatic steatosis. Lipid metabolomics further demonstrated that CPF’s preventive effects on NASH were closely linked to the downregulation of hepatic TG, DG, and Cer levels. Moreover, proteomics confirmed that CPF promotes hepatic lipid metabolic homeostasis [48]. Thus, lipid homeostasis may represent a key pathway through which flavonoids intervene in obesity and prevent hepatic steatosis. Liver phospholipids, represented by PC and PE lipids, play a role in maintaining very low-density lipoprotein (VLDL) and lipid homeostasis in the liver, effectively preventing abnormal lipid accumulation. The levels of PC and PE, as well as their fatty acid chain composition—particularly PC and PE enriched with arachidonic acid chains—are crucial for regulating VLDL secretion. VLDL serves as the primary carrier for transporting triglycerides from the liver into the bloodstream, and normal PC and PE levels are essential for the proper assembly and secretion of VLDL. In contrast, the accumulation of neutral lipids (such as TG, DG, MG, ChE, etc.) is a major contributor to hepatic steatosis. Excessive lipids and their metabolic intermediates (e.g., DG, Cer) can trigger stress responses (such as ER stress), activate inflammatory pathways, and lead to hepatocyte damage and death, thereby driving disease progression from simple steatosis to non-alcoholic steatohepatitis (NASH) [49]. The findings of this study indicate that ABFM intervention significantly increases the expression levels of PC species (e.g., PC(8:0e/13:0), PC(19:0/20:3)) and PE species (e.g., PE(17:0/20:3)), while reducing the expression levels of neutral lipids such as DG(18:3/22:6), TG(12:1e/6:0/12:3), and TG(16:1/18:2/18:2). Additionally, the accumulation of sphingolipids (e.g., Cer) poses multiple risks. Ceramide lipids, a known lipotoxic molecule among sphingolipids, can interfere with insulin signaling pathways and exacerbate hepatic and systemic insulin resistance, which is one of the primary drivers of non-alcoholic fatty liver disease (NAFLD) [49]. Cross-sectional studies on hepatic lipid composition in obesity-related diseases have revealed that the composition (rather than the mere quantity) of hepatic lipid accumulation may be a critical factor in metabolic dysfunction and disease progression. In NAFLD patients, the proportions of palmitic acid (C16:0) and oleic acid (C18:1) in hepatic TG and DG—classified as saturated fatty acids (SFA) and monounsaturated fatty acids (MUFA)—were elevated, while stearic acid (C18:0) was reduced. Long-chain polyunsaturated fatty acids (PUFAs) such as arachidonic acid (AA) and docosahexaenoic acid (DHA) were decreased in TG and phospholipids, with an elevated n−6/n−3 ratio (indicating a pro-inflammatory state) [50]. Furthermore, spatial lipidomics results indicated that dysregulated sphingolipid metabolism is a core feature of hepatic fibrosis and exhibits cell-type specificity (e.g., hepatic stellate cells). Lipid profiling and transcriptomic analysis of fibrotic regions revealed that a high-fat diet significantly upregulated pathways related to sphingolipid metabolism (e.g., serine palmitoyltransferase gene SPTLC2, ceramide synthase genes CERS5/6) and glycosphingolipid synthesis (e.g., A4GALT). Sphingomyelin SM(34:1) exhibited high colocalization with fibrotic regions [51]. In this study, ABFM intake significantly downregulated sphingolipid metabolism pathways and sphingolipid metabolic signaling functional pathways, by affecting the upstream sphingomyelin SM(d18:1/18:0), and it significantly downregulated the expression levels of ceramides Cer(d18:1/25:0), Cer(d18:1/18:0), and Cer(d18:1/16:0). Combined with Masson staining results of liver sections, these findings confirm that ABFM exerts a protective effect against high-fat diet-induced hepatic fibrosis damage.

## 5. Conclusions

As the core of energy metabolism in the organism, regulating hepatic lipid metabolism is a crucial mechanism for alleviating obesity and related metabolic disorders. In summary, ABFM supplementation ameliorated obesity and hepatic lipid metabolism abnormalities in diet-induced obese mice, exerting positive effects on various physiological and biochemical responses. By modulating and improving hepatic lipid metabolism and lipid accumulation in hepatocytes, ABFM effectively regulated and corrected metabolic abnormalities such as dyslipidemia, liver dysfunction, and BAT deformation caused by diet-induced obesity. Additionally, it reversed potential liver damage associated with obesity by ameliorating sphingolipid metabolism dysregulation and hepatic lipid composition, significantly improving the progression of obesity and related metabolic complications. This study further confirmed that ABFM, within a certain dosage range, significantly enhanced hepatic lipid metabolism levels, reversed abnormal hepatic lipid metabolic profiles in mice fed a high-fat diet, and maintained hepatic lipid metabolic homeostasis. The findings demonstrate that red adzuki bean flavonoids, as dietary polyphenols of plant origin, possess potential for preventive and interventional applications in overweight and obesity management. This provides a broader and deeper theoretical foundation for the development of related functional foods and the exploration and application of plant-derived anti-obesity bioactive compounds.

## Figures and Tables

**Table 1:** Flavonoid composition in adzuki beans.

Flavonoids	DZN	GSN	GTE	RTN	DZI	APG	AGL	NGE	Q3G	LQG	VTX	TXF	IVX	KFL
Proportion (%)	32.75 ± 5.34	23.33 ± 5.33	22.03 ± 2.00	10.42 ± 6.69	5.35 ± 0.64	2.21 ± 0.73	1.34 ± 0.46	1.23 ± 0.28	0.71 ± 0.45	0.38 ± 0.09	0.12 ± 0.01	0.05 ± 0.07	0.05 ± 0.02	0.02 ± 0.02

Data are presented as the mean ± standard deviation (*n* = 3). In the table, DZN stands for daidzein; GSN for genistin; GTE for genistein; RTN for rutin; DZI for daidzin; APG for apigenin; AGL for astragalin; NGE for naringenin; Q3G for quercetin 3-glucoside; LQG for liquiritigenin; VTX for vitexin; TXF for taxifolin; IVX for isovitexin; KFL for kaempferol.

**Table 2:** Effects of ABFM supplementation on the mass of various organs in mice.

Group (g)	Liver	Epididymal Fat	BAT	Kidney
M	0.86 ± 0.07	1.53 ± 0.45	0.16 ± 0.14	0.33 ± 0.02
IG-L	0.87 ± 0.10 ^ns^	0.77 ± 0.15 ***	0.07 ± 0.05	0.30 ± 0.05 ^ns^
IG-M	0.96 ± 0.12 ^ns^	0.99 ± 0.26 **	0.11 ± 0.07	0.32 ± 0.04 ^ns^
IG-H	1.09 ± 0.07 ***	0.93 ± 0.17 **	0.15 ± 0.10	0.33 ± 0.02 ^ns^
AC	0.93 ± 0.05 ^ns^	1.09 ± 0.17 *	0.10 ± 0.07	0.33 ± 0.02 ^ns^
N	0.81 ± 0.08 ^ns^	0.65 ± 0.06 ****	0.04 ± 0.03	0.25 ± 0.03 ^**^

Data are presented as the mean ± standard deviation (*n* = 6). In the figure: “*” indicates the level of statistical significance compared to the M group: ^ns^, not significant; *, *p* < 0.05; **, *p* < 0.01; ***, *p* < 0.001; ****, *p* < 0.0001.

## Data Availability

The original contributions presented in the study are included in the article, further inquiries can be directed to the corresponding authors.
